# A prototype bioreactor for the water retting of hemp bast fiber: dynamics of the liquor bacterial consortium across sequential retting cycles

**DOI:** 10.3389/fpls.2026.1856675

**Published:** 2026-08-05

**Authors:** Sara Del Duca, Antonella Lamontanara, Luigi Orrù, Marco Errani, Roberto Reggiani, Vita Maria Cristiana Moliterni

**Affiliations:** 1 Consiglio per la ricerca in agricoltura e l'analisi dell'economia agraria (CREA), Centro di ricerca Genomica e Bioinformatica, Fiorenzuola D'Arda, Italy; 2 Sylfib s.r.l., Milano, Italy; 3 STUARDLAB, San Pancrazio, Italy

**Keywords:** 16S rRNA metabarcoding, industrial hemp, microbial consortium, retting enzymes, sustainable textiles, water retting

## Abstract

The proper extraction of fibers from the surrounding tissues (retting) is a prerequisite for the textile and technical valorization of bast fibers. Fiber retting is naturally carried out by environmental microflora (microbial retting). Among microbial retting, water retting is considered the most effective in producing high-quality textile fiber from bast fiber crops. On the other hand, traditional water retting system resulted unsuitable for the industrial production of textile fiber, due to its environmental, economic and social concerns. A prototype bioreactor has been developed, based on a patented process, for the water retting of hemp bast fibers, overcoming the major constraints of the traditional method. The bacterial succession occurring in the retting liquor when a portion (5%) of the liquor is recycled has been studied across eight subsequent retting cycles. Retting liquor has been sampled before process starts, mid-process and endpoint of each cycle. 16S rRNA metabarcoding and functional predictions along the subsequent retting cycles gave back a succession resembling what has been observed in conventional retting ponds during a single retting cycle. The negative impact of liquor recycling on the predicted enzyme function abundances has been measured from the third retting cycle. Useful information was obtained on the possible valorization of liquor waste in sustainable agricultural practices.

## 1 Introduction

The transition towards climate neutrality and the need for more sustainable productions has increased interest in raw materials from renewable natural sources. Bast fiber crops, like flax, hemp, ramie and kenaf can produce raw materials for textiles, pulp and construction industries also providing ecosystem services, depending on the species, cropping system and processing techniques used. Bast fibers are strong, light, less expensive, and renewable, so they could be a sustainable alternative to synthetic fibers for textile and technical applications (Rangappa et al., 2022).

Hemp fiber, as well as flax, has been used for textiles for thousands of years by ancient Asian and European communities (Clarke, 2010) but in the late 18^th^ century it has been progressively substituted by cotton due to the mechanization of its spinning system, and in the early 20^th^ century, by the less expensive synthetic fibers (Angulu and Gusovious, 2024). The legal ban of hemp cultivation due to its psychoactive properties in the most of European countries and in the United States during the mid-20^th^ century created a gap into the hemp processing lines (Zimniewska, 2022) that now needs to be gradually filled to enable the effective reintroduction of hemp fiber into the textile industry. Low impact processing techniques and products standardization are the most relevant challenges for establishing a sustainable and profitable production of bast fiber-based textiles (Angulu and Gusovius, 2024).

Industrial hemp (delta−9−tetrahydrocannabinol, THC, content below 0.2%) is characterized by a low-input cultivation requirements and a high, multipurpose biomass yield (Finnan and Burke, 2013) with versatile utilization pathways (Schluttenhofer and Yuan, 2017). Suitable for supporting regenerative agriculture practices, industrial hemp has the potential to deliver positive environmental impact, not only through soil restructuring and fertilization (Adesina et al., 2020), but also through climate change mitigation thanks to its high CO_2_ fixation rate under low nitrogen input (Tang et al., 2017). Most of the fixed carbon is stored in the harvested stem and, ultimately, in durable products such as textiles or construction materials (Adesina et al., 2020), making industrial hemp one of the more interesting crops in contributing to the European Green Deal objectives (European Commission, 2024).

Hemp stalk is composed of a *cortical region* comprising the epidermis, cortical parenchyma, endoderm and phloem, and a *medullary region* composed of xylem and medullary parenchyma. When mature, the pith is hollow and the two stem components mentioned above can be mechanically separated, yielding what are known as *bark*, where textile fibers are located, and the *shives* (or hurds), which account for approximately 20-25% and 75-80% of the hemp stalk, respectively (Zimniewska, 2022). Bast fibers are associated to the phloem and grouped in fiber bundles through their middle lamellas which, together with primary cell walls, are particularly rich in pectins (Sorieul et al., 2016). The physical-chemical and technical properties of hemp fibers are strongly dependent on plant genetics, growing conditions, harvesting techniques and extraction method used (Mussig and Martens, 2003; Shahzad, 2012; Graupner et al., 2022; Zimniewska, 2022; Green et al., 2024). Similarly to other bast fibers, hemp fiber is composed of cellulose, hemicellulose, lignin, pectin and waxes (non-cellulosic compounds), cellulose being the main macromolecular compound (40-80% dry weight). The physical characteristics of the fiber mainly depend on cellulose, hemicellulose, and lignin contents, cellulose being the most rigid and strong organic component of the fiber (Shelly et al., 2025). Cellulose is a linear polysaccharide composed of repeating disaccharide units; 30–100 molecules of cellulose aggregate to form an elementary fibril. The combination of 10–50 elementary fibrils and hemicellulose forms micro-fibrillated cellulose units which are assembled with non cellulose components in the cell wall of each elementary fiber (Lyu et al., 2021). Pectins are a complex set of polysaccharides; the most relevant is galacturonic acid, which glues together microfibrils belonging to different elementary fibers, producing fiber bundles. Pectins confer flexibility to hemp fibers but negatively affect their downstream textile and technical valorization (Lyu et al., 2021; Zimniewska, 2022). Hemp elementary fibers can be up to 55 mm long, while fiber bundles can reach lengths of up to 3000 mm (Zimniewska, 2022).

To be used for textile or technical applications, hemp fibers must be extracted from the surrounding tissues and individualized; the higher the degree of fiber individualization, the finer the resulting fibers and the greater their potential for valorization in high quality textiles. Retting is the process that leads to the degradation of pectins, hemicelluloses and lignin, resulting in the loosening of the stem structure (i.e., separation of the bark from the woody core) and, depending on the nature of the starting biomass, the method and the duration, to the progressive individualization of the bast fiber bundles (Angulu and Gusovius, 2024). Several retting methods have been extensively reviewed in literature (Paridah et al., 2011; Lee et al., 2020; Lyu et al., 2021; Angulu and Gusovius, 2024), and those considered more suitable for the extraction of textile fibers are *microbial retting*, *enzymatic retting*, *chemical retting* and *physical retting* (reviewed in Zimniewska, 2022), based on the nature of the strength used to carry out the process.

*Microbial retting* is the most natural and ancient method to extract hemp bast fibers, as it relies on the digestion of pectic substances and non-cellulosic materials by microorganisms naturally living in the environment. This method is referred to as water retting when the straws are submerged in water, and as dew retting when they are laid on the soil and exposed to natural environmental conditions. Dew retting largely relies on the polygaracturonase and hemicellulase activity of indigenous soil fungi colonizing hemp straws. Water retting is mainly carried out by anaerobic pectinolytic bacteria which are strongly selected in retting ponds (Paridah et al., 2011). Microbial retting methods require 30–45 days but their products are quite different: dew retted fibers are often coarse and of heterogeneous quality; conversely water retting significatively increases yield and quality of the retted fibers, being considered the most suitable for textile downstream applications (Paridah et al., 2011; Lee et al., 2020; Lyu et al., 2021; Zimniewska, 2022; Różańska et al., 2023; Angulu and Gusovius, 2024). On the other hand, both methods raise some environmental and social concerns: dew retting alters the natural soil microbiota, negatively affecting agroecological balance (Angulu and Gusovius, 2024); water retting, due to its high water demand, environmental pollution with anaerobic and potentially toxic bacteria, and the unhealthy working conditions for operators, also poses significant issues (Paridah et al., 2011; Angulu and Gusovius, 2024).

Over the past twenty years, several research efforts have focused on innovating water retting technology, focusing on the ecological, economic, and social sustainability of the process to enable the stable integration of hemp fiber into the textile industry (Zimniewska, 2022). Among these initiatives is the development of an innovative water retting technology, based on a patent (Tofani and Errani, 2006) together with the related prototype bioreactor in which the process is carried out. The bioreactor is filled with spring water and is set up to carry out the water retting of hemp bast fiber within three days, without any kind of additive. Retting liquor is partially recycled in subsequent retting cycles, reducing water exploitation.The study of the bacterial retting consortium operating into the prototype bioreactor is an integral part of process characterization in view of its application to industrial production. Therefore, a set of eight subsequent retting cycles have been carried out within the bioreactor, where retting liquor has been sampled before process starts, mid-process, and endpoint. The experimental design was also devised to assess the impact of partial liquor recycling on the retting community across consecutive cycles. A targeted metagenomic approach has been used as elective method to describe bacterial communities and their succession in complex samples, enabling the investigation of community structure, taxonomic composition and the predicted enzyme functions reservoir (Zhao et al., 2016; Law et al., 2020; Djemiel et al., 2020; Fu et al., 2024). Retting liquor waste has been analyzed to verify its possible reuse as organic fertilizer in sustainable agricultural practices. Indeed, as with effluents of olive oil production or pig slurry, effluents from hemp water retting ponds can also be applied to agricultural soil, once their environmental impact has been evaluated (Castaldini et al, 2001). Fertilization experiments demonstrated that water from natural hemp retting ponds had no negative effects neither on soil microflora nor on morpho-biometric traits and yield of wheat crop (Castaldini et al., 2001).

## 2 Materials and methods

### 2.1 The prototype bioreactor

**Figure 1:**
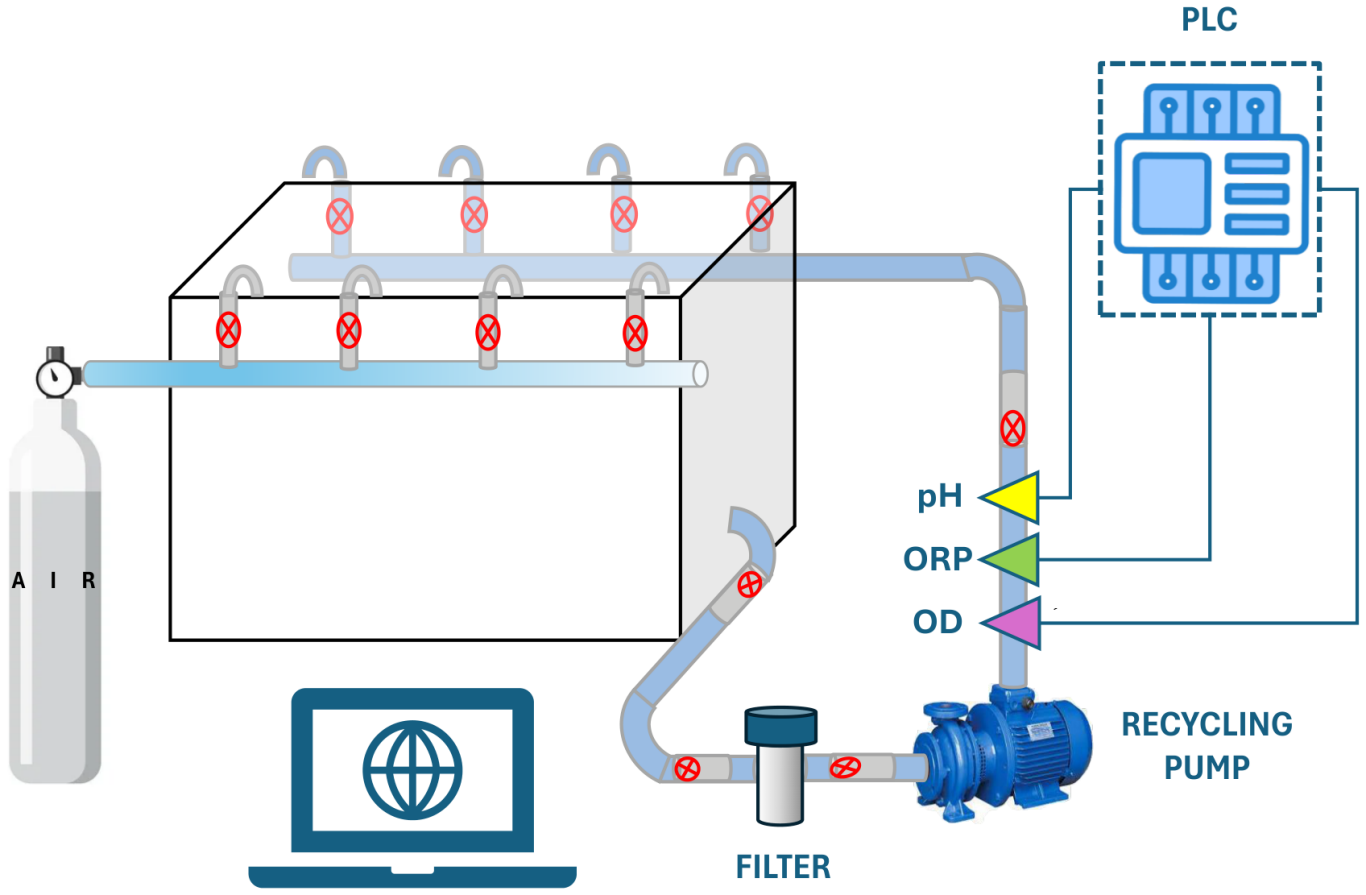
Schematic representation of the prototype bioreactor.

The bioreactor open tank, realized within STUARDLAB laboratories and schematized in **Figure 1**, has a capacity of 200 l and is equipped with a mild warming system to keep the water temperature above 20 °C. Air diffusers allow air injection within the tank, and a water recycling system contributes to the homogenization of the retting environment, reducing water waste. pH, redox potential (ORP) and Dissolved Oxygen sensors (DO), with integrated temperature probe, are distributed downstream to the water recycling pump; all sensors are connected to a PLC (Programmable Logic Controller). A sediment filter (mechanic) is placed upstream to the recycling pump to preserve the recycling pump from biomass debris accumulation. The core of technology is enclosed in a proprietary software, able to control and direct the retting process, based on the integration of process parameters which are continuously measured and stored by a data logger. The bioreactor is filled with spring water to avoid chlorine contamination and can process up to 4 kg of decorticated hemp fiber (hemp bark), which is arranged in separate layers within an iron loading cage, operated by an electronic winch. To be processed into the bioreactor, harvested hemp straws are briefly air-dried and decorticated, separating the bast from the woody core. This step reduces the biomass volume by approximately 75%, thereby lowering both spring water consumption and time required for fiber extraction.

### 2.2 Experimental design and sampling

**Figure 2:**
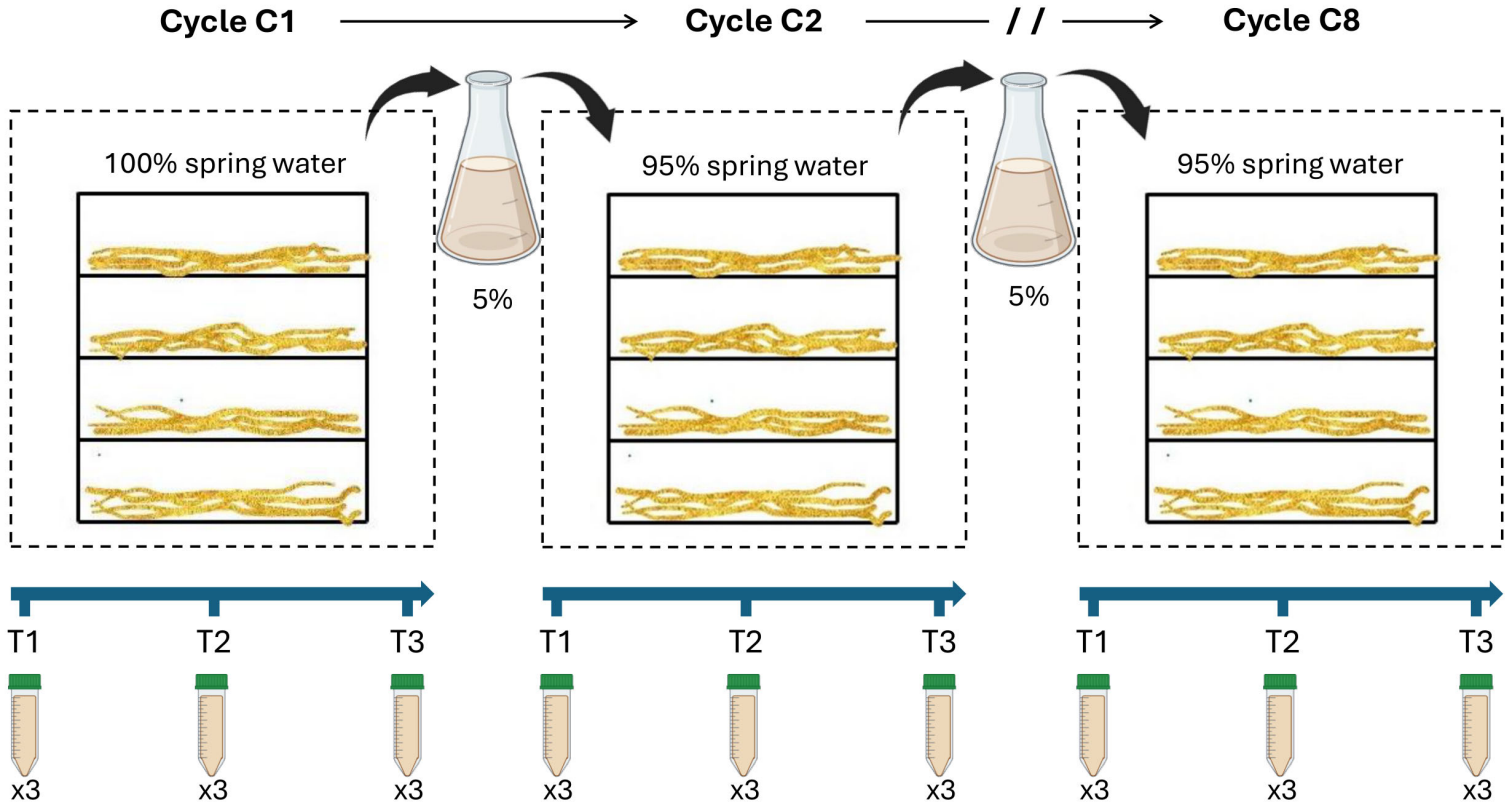
Schematic representation of the experimental strategy. Retting cycles C3-C7 are omitted. Figure partially created with BioRender.com.

Bast fiber was sourced by Italian monoecious fiber hemp crops, that were mechanically harvested, air-dried, and industrially decorticated. All the retting cycles were carried out using the same source of spring water and different batches of hemp bast fiber, following the retting protocol previously established by the technology owner, as follows: 4 kg of hemp bast fibers were arranged in layers within the iron loading cage and submerged into the bioreactor, previously filled with 200 l of spring water. Before starting retting (T1), 50 ml of retting liquor was sampled in triplicate in sterile vials and stored at -20 °C until DNA extraction. Based on the total cycle duration, estimated by the proprietary software from the process physical-chemical parameters, a second liquor sampling (50 ml, in triplicate) was performed after 35 h (T2), and a third sampling at 70 h (T3; end process). Eight subsequent retting cycles have been carried out; from the second retting cycle onward, 5% of the retting liquor was diluted in fresh spring water and recycled in the successive retting cycle (**Figure 2**). The average duration of each cycle was 70 h:07 min (± 1 h:43 min). At the end of the eighth retting cycle, liquor waste (1000 ml, in triplicate) was sampled for analysis to determine its chemical-physical composition. Fiber produced by the eight subsequent retting cycles (4 kg dry bast fiber per cycle), after washing and drying has been further processed through a small-scale wool spinning line until the production of 100% hemp yarn, up to a titer of 6 Nm (metric Number) and 9Nm (**Supplementary Figure 1**).

### 2.3 Analysis of retting liquor waste composition

Retting liquor waste has been analyzed by an external laboratory (Area Laboratorio Prove e Misure, SSICA - Parma, Italy) accredited by ACCREDIA in accordance with the UNI CEI EN ISO/IEC 17025 standard. Chemical-physical parameters and methods used for analysis are listed in **Table 1**.

**Table 1:** List of measured parameters, methods and techniques used for retting liquor waste analysis.

Parameter	Method	Technique
pH	APAT CNR IRSA 2060 Man 29 2003	Potentiometry
Total Suspended Solids	APAT CNR IRSA 2090 B Man 29 2003	Gravimetry
Settleable solids	APAT CNR IRSA 2090 C Man 29 2003	Gravimetry
Chemical Oxygen Demand (COD)	ISO 15705:2002	UV-VIS spectrophotometry
Biological Oxygen Demand (BOD5)	LEI/SSICA 1995	UV-VIS spectrophotometry
Nitrate	APAT CNR IRSA 4020 Man 29 2003	Ion chromatography
Nitrite	APAT CNR IRSA 4020 Man 29 2003	Ion chromatography
Ammoniacal Nitrogen	LEI/MP/N.13–2017 Rev. 9	UV-VIS spectrophotometry
Total Nitrogen	LEI/SSICA 2009/4	UV-VIS spectrophotometry.
Phosphorus (PO4-P)	LEI/MP/N.12–2017 Rev. 9	UV-VIS spectrophotometry
Chloride	APAT CNR IRSA 4020 Man 29 2003	Ion chromatography
Sulphate	APAT CNR IRSA 4020 Man 29 2003	Ion chromatography

### 2.4 DNA extraction, sequencing and bioinformatics

DNA was extracted from 25 ml of pelleted retting liquor samples (9000 g, 8 °C, 20 min) through the NucleoSpin Soil Mini Kit (Macherey-Nagel), adapting the manufacturer’s instructions as follows: pellet has been resuspended in 700 µl of SL1 Buffer and transferred to a Nucleospin bead tube type A, then 150 µl of Enhancer SX have been added to each tube before vortexing. 1 µl of each DNA eluted in 30 µl of SE buffer has been quantified by NanoDrop™ 2000 (Thermo Scientific) and verified on 1% agarose gel. All the samples were further quantified by a Qubit fluorimetric method following the manufacturer’s instructions (Qubit dsDNA HS Assay Kit, Thermo Scientific). Libraries were generated following the Illumina 16S Metagenomic Sequencing Library Preparation protocol (Part # 15044223 Rev.B) targeting the 16S V3-V4 region. Amplicon libraries were mixed with 10% PhiX control library to increase sequence diversity, loaded and sequenced on the Illumina^®^ MiSeq™ instrument, according to the manufacturer’s instructions, generating 300 bp paired end reads. Raw sequence files were deposited in the NCBI Sequence Read Archive (SRA) under BioProject accession PRJNA1453432.

Trimmomatic (version 0.39) (Bolger et al., 2014) was used to remove adapters and low-quality reads using a quality cutoff of 20 in 20 bp sliding windows. Filtered reads were processed using the QIIME2 pipeline (version 2020.8) (Bolyen et al., 2019). Paired-end reads were merged using Pear (version 0.9.11) (Zhang et al., 2014). The QIIME2 DADA2 plugin was used to denoise the sequences, remove chimeras and generate amplicon sequence variants (ASVs) (Callahan et al., 2016). ASVs were clustered into OTUs at 97% identity using QIIME2 vsearch cluster-features-open-reference tool (Rognes et al., 2016). Taxonomy classification was made using the SILVA reference database (version 138_99). Obtained abundance and taxonomy tables were then analyzed through the phyloseq R package (version 1.40.0) (McMurdie and Holmes, 2013). Representative sequences that were not assigned to domain Bacteria were removed from the dataset.

The possible ecological associations among bacterial OTUs were inferred using the SPIEC-EASI algorithm (Kurtz et al., 2015), through the SpiecEasi (version 1.1.3) (Kurtz et al., 2017) R package. To obtain a network for each retting cycle, samples were grouped accordingly and processed. The obtained networks were then compared, inspecting their shape, structural properties and node degree distributions.

### 2.5 Functional predictions

PICRUSt2 (version 2.5.3), a bioinformatic tool that predicts microbial functions in amplicon sequencing data, using a database of annotated reference genomes (Douglas et al., 2020), was applied to depict the functional profile of the retting consortium. Among all enzymes predicted by PICRUSt2, those known to be involved in the retting process of bast fibers (Liu et al., 2017a; Liu et al, 2017b; Zimniewska, 2022) have been selected and their representation within the retting consortium across eight subsequent cycles has been investigated. In particular, these included: i) pectinases, such as EC:3.1.1.11 (pectinesterase), EC:3.2.1.15 (polygalacturonase), EC:4.2.2.2 (pectate lyase), EC:4.2.2.10 (pectin lyase), EC:3.2.1.171 (rhamnogalacturonan hydrolase), EC:4.2.2.23 (rhamnogalacturonan endolyase), EC:3.1.1.6 (pectin acetylesterase), EC:3.1.1.73 (feruloyl esterase), and EC:4.2.2.9 (pectate-disaccharide-lyase); ii) cellulases, such as EC:3.2.1.4 (endoglucanase), EC:3.2.1.91 (cellulose 1, 4-β-cellobiosidase), and EC:3.2.1.21 (β-glucosidase); iii) hemicellulases, such as EC:3.2.1.8 (endo-1, 4-β-xylanase), EC:3.2.1.37 (xylan 1, 4-β-xylosidase), EC:3.2.1.78 (mannan endo-1, 4-β-mannanase), EC:3.2.1.55 (α-L-arabinofuranosidase), EC:3.2.1.23 (β-galactosidase), EC:3.2.1.25 (β-mannosidase), EC:3.2.1.51 (α-L-fucosidase), and EC:3.2.1.177 (α-D-xyloside xylohydrolase); iv) cell wall esterases, such as EC:3.1.1.6 (pectin acetylesterase), EC:3.1.1.73 (feruloyl esterase), and EC:3.1.1.72 (acetylxylan esterase); v) ligninolytic enzymes, such as EC:1.10.3.2 (laccase), EC:1.11.1.13 (manganese peroxidase), and EC:1.11.1.14 (lignin peroxidase).

### 2.6 Statistical testing

Statistical analysis on bacterial community and predicted enzyme functions was performed using the vegan (version 2.6.6.1) (Oksanen et al., 2007) and stats (version 4.4.1) R packages. All the plots were obtained through the ggplot2 R package (version 3.5.1) (Wickham, 2006). To normalize the sequencing depth, abundance data were rarefied before analyses; rarefaction was performed at the minimum sequencing depth among all samples (corresponding to 5, 443 reads per sample). Rarefaction curves are reported in **Supplementary Figure 1**.

Differences in bacterial community structure and predicted enzyme functions were analyzed by principal coordinate analysis (PCoA) based on Bray-Curtis dissimilarity, and the effect of the “retting cycle” and “sampling time” variables was tested on the relative abundances using permutational analysis of variance (PERMANOVA).

Bacterial community diversity was characterized using observed richness, Shannon diversity and evenness indices. The effect of the different sampling time on the diversity indices, and on the absolute abundances of different bacterial orders and predicted enzyme functions was tested using analysis of variance (ANOVA) followed by Tukey HSD *post-hoc* test.

## 3 Results

### 3.1 Retting liquor waste composition

Analysis confirmed retting liquor waste (**Table 2**) to be similar to effluents of olive oil production (Chaâri et al., 2022) in terms of pH, but particularly enriched in organic matter (COD = 3547 mg/l O_2_) and soil nutrients. The low COD/BOD5 ratio (1.47) indicates the high biodegradability of the organic matter contained. Analysis also highlighted retting liquor waste to be a good source of total nitrogen, phosphorus, and sulfur.

**Table 2:** Retting liquor waste composition.

Parameter	Value	Unit
pH	5.3	
Total Suspended Solids	313	mg/l
Settleable solids	0.9	ml/l
Chemical Oxygen Demand (COD)	3547	mg/l O2
Biological Oxygen Demand (BOD5)	2400	mg/l O2
Nitrate	1.7	mg/l
Nitrite	1.2	mg/l
Ammoniacal Nitrogen	24.5	mg/l
Total Nitrogen	79	mg/l
Phosphorus	21.4	mg/l
Chloride	325	mg/l
Sulphate	89	mg/l

### 3.2 Temporal trends of liquor microbiota during sequential retting cycles

#### 3.2.1 Investigation of the retting liquor bacterial community

A total of 380, 702 bacterial 16S rRNA gene reads were generated after quality filtering steps and rarefaction. These high-quality sequences were grouped into 885 bacterial OTUs.

**Figure 3:**
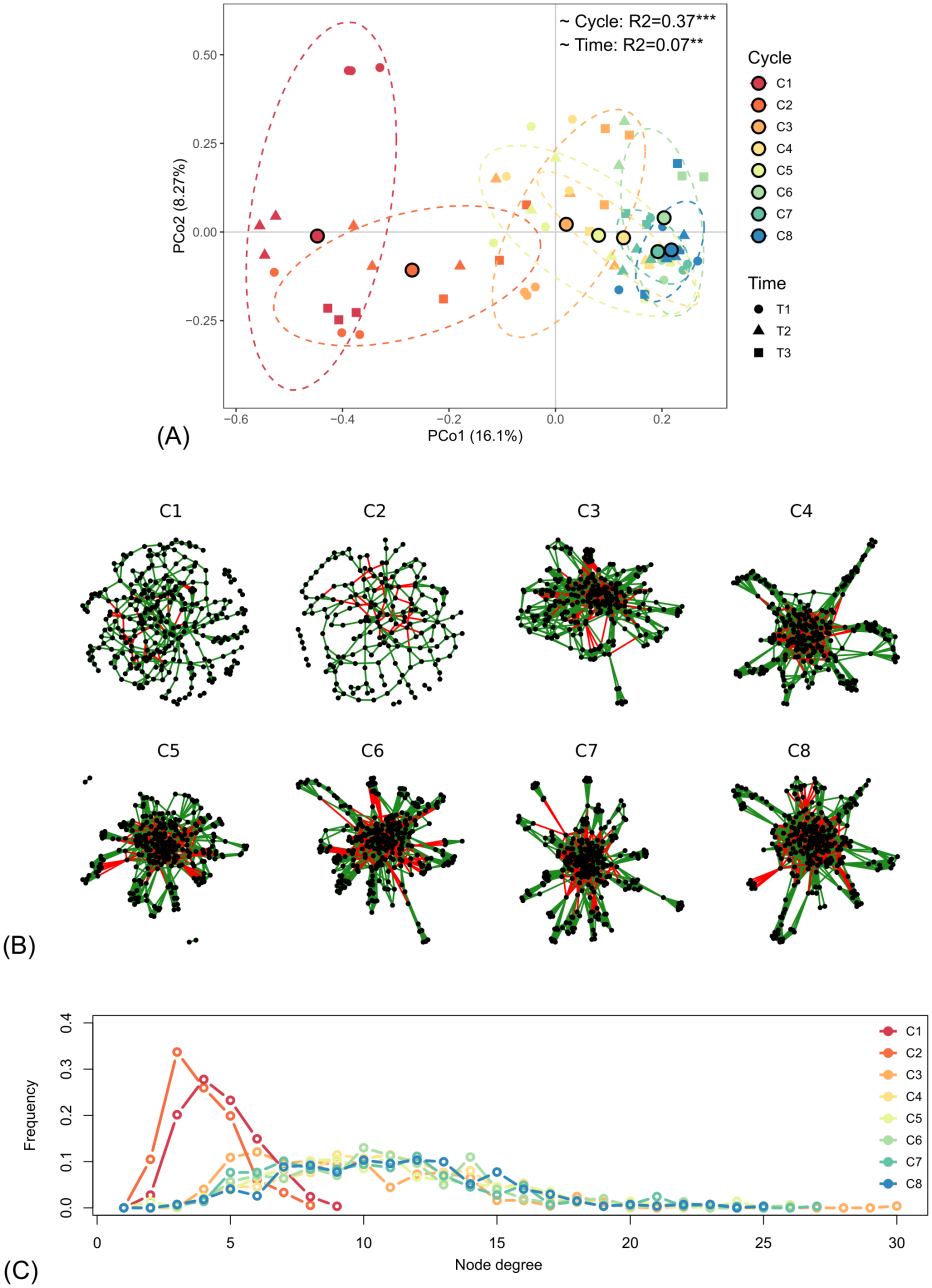
**(A)** PCoA of the liquor bacterial community. Samples (small points without black border) are colored based on the cycle, and the different sampling times are evidenced by different point shapes. The big dots with black borders represent the centroids of the sample groups. Confidence ellipses at 75% are reported with dashed lines. **(B)** Co-occurrence networks obtained by the bacterial community of the distinct retting cycles. Edges are reported in green if the relation between the nodes is positive, and in red if negative. **(C)** Node degree distributions of the obtained networks.

A PCoA analysis was performed to analyze and visualize the changes in bacterial community composition during eight successive retting cycles and at different sampling times (T1, T2, T3). The PCoA showed a transition of the bacterial community structure during the cycles along the first PCoA axis (**Figure 3A**). Specifically, when analyzing all cycles from C1 to C8, a progressive shift in community composition was evident from C1 to C2, extending through the later experimental groups. In cycles C6-C8, the samples appeared more intermixed despite being derived from different retting cycles. Accordingly, the influence of the “cycle” variable on the bacterial community structure varied depending on the set of cycles considered: across the full sample set (C1-C8), the “cycle” variable explained 37% of the variance (PERMANOVA R^2^ = 0.37, p-value<0.001). In the subset C3-C8, the effect decreased to a moderate level (PERMANOVA R^2^ = 0.22, p-value<0.001). In cycles C6-C8, a further reduction in the impact of the “cycle” variable on the bacterial community was observed (PERMANOVA R^2^ = 0.13, p-value=0.02), indicating limited separation between groups. Also, along the eight cycles a reduction of variability in the bacterial composition across samples within each cycle, as highlighted by the gradual reduction of major axis of the confidence ellipses (**Figure 3A**).

Possible differences in ecological associations among bacterial OTUs across the successive retting cycles were investigated using co-occurrence networks. In these networks, each node represents a bacterial OTU, and an edge between two nodes indicates that their abundances are not conditionally independent, implying a linear association between the corresponding taxa (Kurtz et al., 2015). All networks contained a comparable number of nodes but showed marked differences in connectivity. Networks obtained from cycles C1 and C2 showed a substantially different network conformation (**Figure 3B**) characterized by a lower number of edges and a higher proportion of positive relative to negative associations (**Table 3**), indicating reduced interaction. These networks also exhibited larger diameters, as indicators of a sparse and weakly connected structure, and a peaked degree distribution (**Figure 3C**), reflecting the predominance of low-degree nodes.

**Table 3:** Structural properties of the obtained networks.

Cycle	Nodes	Total edges	Positive edges	Negative edges	Diameter
C1	288	524	484	40	19
C2	181	262	226	36	16
C3	248	1049	791	258	7
C4	262	1293	1074	219	8
C5	284	1397	1017	380	7
C6	300	1472	1102	370	8
C7	287	1367	1041	326	8
C8	271	1376	1003	373	7

The total number of nodes for each network is reported, as well as the total number of edges, separated based on the positivity or negativity of their relation, and the diameter of the networks.

**Figure 4:**
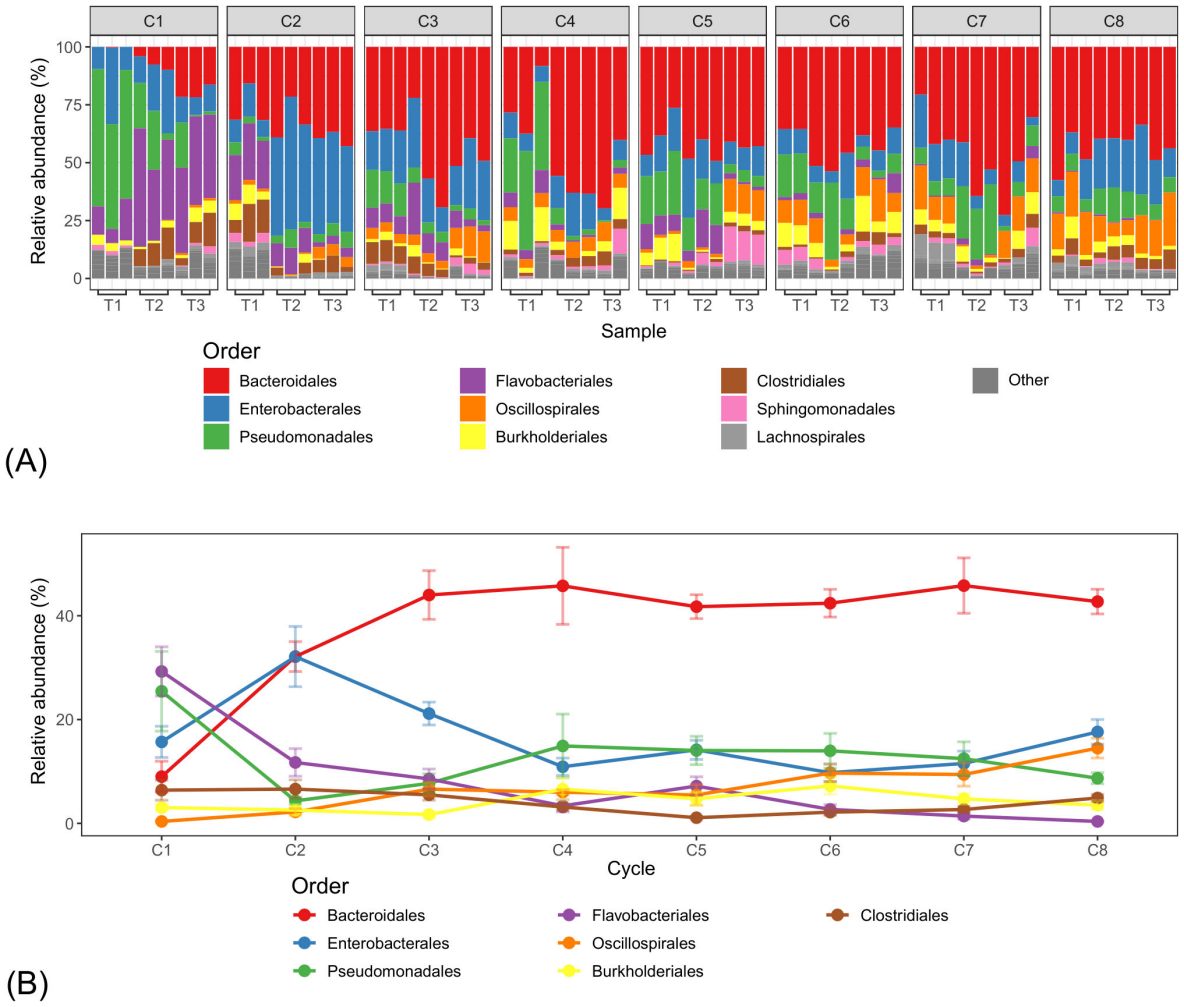
**(A)** Taxonomic composition at the order level of liquor consortium from subsequent retting cycles (C1-C8) and sampling times (T1, T2, T3). Orders representing <6% of the whole community have been reported as “Other”. **(B)** Average relative abundances of the most abundant bacterial orders across cycles. Error bars represent the standard error. Different orders are shown in distinct colors.

The temporal shift in the liquor bacterial community was evident when inspecting the taxonomic composition of the samples at the phylum, order and genus levels (**Figures 4A, B**; **Supplementary Table 1**; **Supplementary Figure 3**). Taxonomic classification revealed the retting consortium to be mainly composed of Bacteroidota, Proteobacteria, and Firmicutes at variable relative abundances, depending on the retting cycle and time of sampling. Bacteroidota showed a slight increase in their relative abundance, from 39.3% in cycle C1 to 43.2% in C8 whereas Firmicutes increased more markedly (from 11.0% to 24.3%), with this latter change being statistically significant (**Supplementary Table 1**). Conversely, Proteobacteria showed a general (albeit non-significant) reduction, starting from a relative abundance of 49.6% in C1 to 32.2% in C8. At the order level, from C1 to C8 a significant increase in bacteria belonging to the orders Bacteroidales (from 9.06% to 43.2%) and Oscillospirales (from 0.39% to 14.7%) has been observed, as well as a significant decrease in bacteria belonging to the order Flavobacteriales (from 29.4% to 0.38%) and, to a lesser extent, Pseudomonadales (from 25.5% to 8.78%) and Clostridiales (from 6.43% to 4.95%). Bacteria belonging to the orders of Enterobacterales and Burkholderiales showed fluctuating relative abundances within the consortium along C1-C8 cycles, being Enterobacterales more represented than Burkholderiales. Furthermore, Enterobacterales and Pseudomonadales, after showing a drastic opposite trend within C1 and C4, seem to fluctuate in their relative representations within the retting consortium between C5 and C8 cycles, with a mild opposite trend (**Figure 4B**). Additionally, a general stabilization in the composition of bacterial community became apparent starting from experiments C5 and C6. The investigation of the bacterial genera more represented within the most abundant orders revealed that different genera often followed very different temporal trends, compared to the global trend of the whole order (**Supplementary Figure 3**). Within Bacteroidales, only the genus *Bacteroides* showed an increase in its relative abundance in cycles from C1 to C3, with a successive stabilization during the following cycles, accounting for the global increase of the whole order. Among Enterobacterales, the genera *Escherichia-Shigella* mainly contributed to the genus relative abundance, as well as *Caproiciproducens* and *Anaerofilum* within the order of Oscillospirales, and *Fonticella* within the order of Clostridiales. On the other hand, the genus *Cloacibacterium* accounted for the drastic temporal reduction in abundance of the Flavobacteriales, and many genera accounted for the reduction of Clostridiales relative abundance along subsequent retting cycles. Finally, the genera *Enterobacter* (Enterobacterales), *Pseudomonas* and *Acinetobacter* (Pseudomonadales) exhibited pronounced fluctuations across subsequent cycles, without displaying a clear temporal trend.

#### 3.2.2 Functional predictions

**Figure 5:**
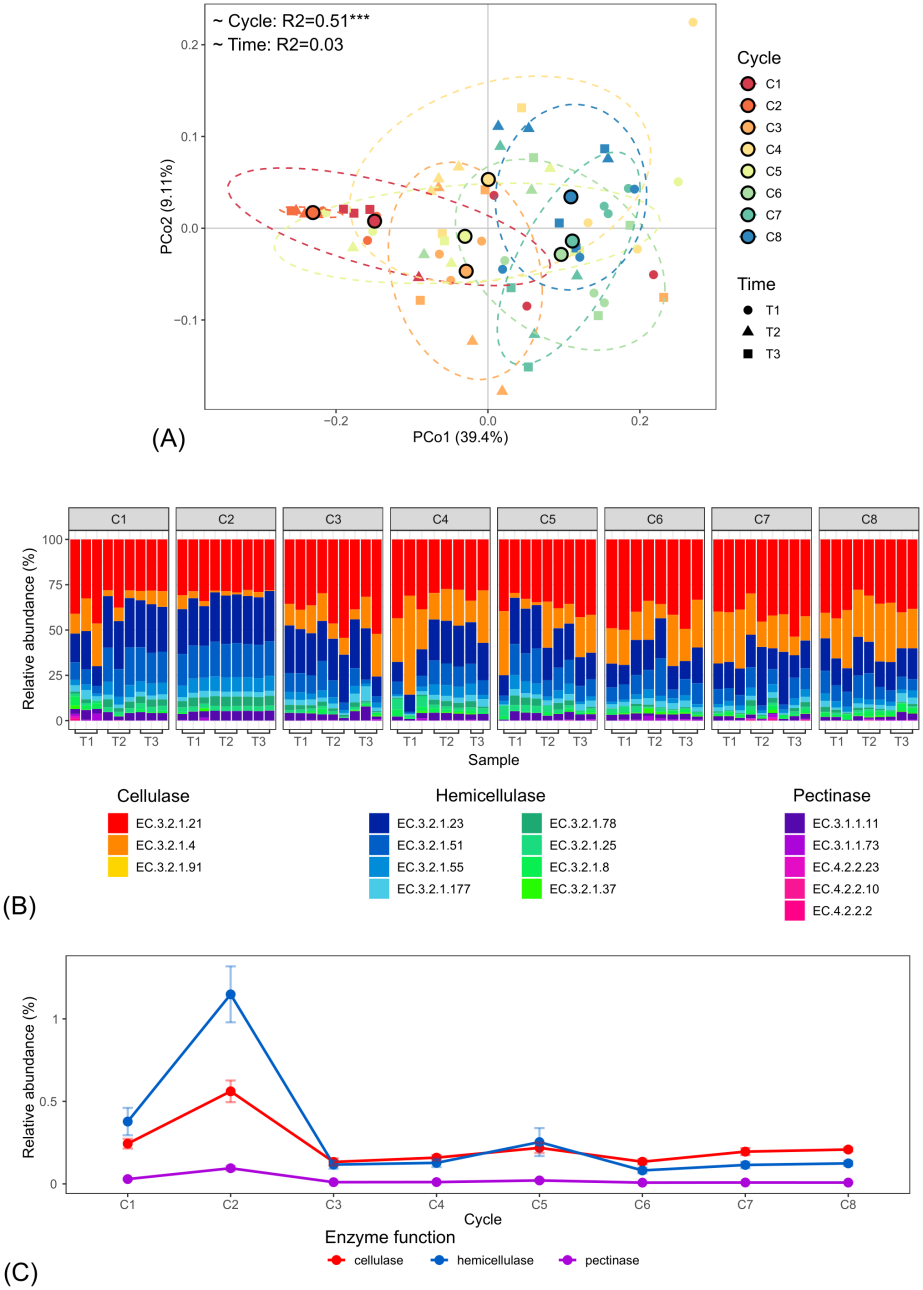
**(A)** PCoA of the predicted retting enzyme function composition. Samples (small points without black border) are colored based on the cycle, and the different retting phases are evidenced by different point shapes. The big dots with black borders represent the centroids of the sample groups. Confidence ellipses at 75% are reported with dashed lines. **(B)** Predicted enzyme function composition of liquor from different cycles (C1-C8) and sampling times (T1, T2, T3). Bars represent the relative abundances within the retting−enzyme subset (i.e., normalized to 100%). **(C)** Average relative abundances of the retting enzyme function guilds calculated over the total predicted enzyme function pool across cycles. Error bars represent the standard error. Different enzyme guilds are shown in distinct colors.

A total of 1, 980 enzyme functions have been predicted using PICRUSt2 starting from the amplicon sequencing data. Among them, 16 enzyme functions considered relevant to the retting process were identified (see Materials and Methods) and their relative abundances within the dataset have been analyzed across subsequent cycles. Five pectinases, eight hemicellulases and three cellulases have been identified (**Table 4**), accounting on average for 0.6% of the total enzymatic functions predicted across samples. As already observed for the total bacterial community, the PCoA showed a transition in the composition of the retting-related enzyme functions during the cycles along the first PCoA axis (**Figure 5A**). A shift was observed from the C1-C2 samples group, extending to C3-C4-C5 and finally to the C6-C7-C8 cluster. Also in this case, the magnitude of the influence of the “cycle” variable on the enzyme function composition varied depending on the subset of cycles considered: across the full sample set (C1-C8), the “cycle” variable explained a substantial proportion of the variance (PERMANOVA R^2^ = 0.51, p-value<0.001). In the subset C3-C8, the effect decreased to a moderate level (PERMANOVA R^2^ = 0.19, p-value=0.01). In the C6-C8 subset, the effect remained similar (PERMANOVA R^2^ = 0.19, p-value=0.02), indicating a consistently moderate but significant influence of the cycle on enzyme function profiles.

**Table 4:** Enzyme functions relevant to fiber retting predicted in our dataset.

ID	Description	Guild	CAZy family
EC:3.1.1.11	Pectinesterase	Pectinase	CE8
EC:3.1.1.73	Feruloyl esterase	Pectinase	CE1
EC:3.2.1.177	α-D-xyloside xylohydrolase	Hemicellulase	GH31, GH120
EC:3.2.1.21	β-glucosidase	Cellulase	GH1, GH3
EC:3.2.1.23	β-galactosidase	Hemicellulase	GH1, GH2, GH35, GH42
EC:3.2.1.25	β-mannosidase	Hemicellulase	GH2, GH5
EC:3.2.1.37	Xylan 1, 4-β-xylosidase	Hemicellulase	GH3, GH43
EC:3.2.1.4	Endoglucanase	Cellulase	GH5, GH9, GH12, GH45
EC:3.2.1.51	α-L-fucosidase	Hemicellulase	GH29, GH95
EC:3.2.1.55	α-L-arabinofuranosidase	Hemicellulase	GH43, GH51, GH5
EC:3.2.1.78	Mannan endo-1, 4-β-mannosidase	Hemicellulase	GH5, GH2
EC:3.2.1.8	Endo-1, 4-β-xylanase	Hemicellulase	GH10, GH1
EC:3.2.1.91	Cellulose 1, 4-β-cellobiosidase	Cellulase	GH6, GH7
EC:4.2.2.10	Pectin lyase	Pectinase	PL1
EC:4.2.2.2	Pectate lyase	Pectinase	PL1, PL3
EC:4.2.2.23	Rhamnogalacturonan endolyase	Pectinase	PL4, PL11

Analysis of the predicted enzyme functions composition revealed that the fraction of the total enzymatic pool associated with the retting process was dominated by the cellulases EC:3.2.1.21 (β-glucosidase) and EC:3.2.1.4 (endoglucanase), together with the hemicellulases EC:3.2.1.23 (β-galactosidase) and EC:3.2.1.51 (α-L-fucosidase). Pectinases, although detected in all samples, accounted for only a minor proportion of the retting enzymatic pool (**Supplementary Table 2**). A temporal trend for some of the enzyme functions investigated was observed (**Figures 5B, C**; **Supplementary Table 2**). More specifically, the hemicellulase EC:3.2.1.23 (β-galactosidase) showed an initial significant increase from cycle C1 (in which it represented the 0.17% of the total enzymes) to C2 (0.48%), followed by a significant decrease in cycle C3 (0.06%), after which its abundance remained stable through C8. The same trend was observed also for the hemicellulases EC:3.2.1.51 (α-L-fucosidase), EC:3.2.1.55 (α-L-arabinofuranosidase), EC:3.2.1.177 (α-D-xyloside xylohydrolase), EC:3.2.1.78 (mannan endo-1, 4-β-mannosidase) and EC:3.2.1.25 (β-mannosidase), as well as for the cellulase EC:3.2.1.21 (β-glucosidase) and for the pectinase EC:3.1.1.11 (pectinesterase). On the other hand, the cellulase EC:3.2.1.4 (endoglucanase), showed a significant increase in its abundance (from a total of 0.05% in C1 to 0.09% in C8). The plot of the summarized enzymatic guilds (**Figure 5C**) revealed a comparable trend in cellulase, hemicellulase, and pectinase activities across cycles C1 to C8 in the retting consortium. It also highlighted that cycle C2 was characterized by the highest relative abundance of retting enzyme functions over the total enzymatic pool, as well as by the highest hemicellulase-to-cellulase ratio.

### 3.3 Effect of the sampling time on the retting consortium and predicted enzyme functions

**Figure 6:**
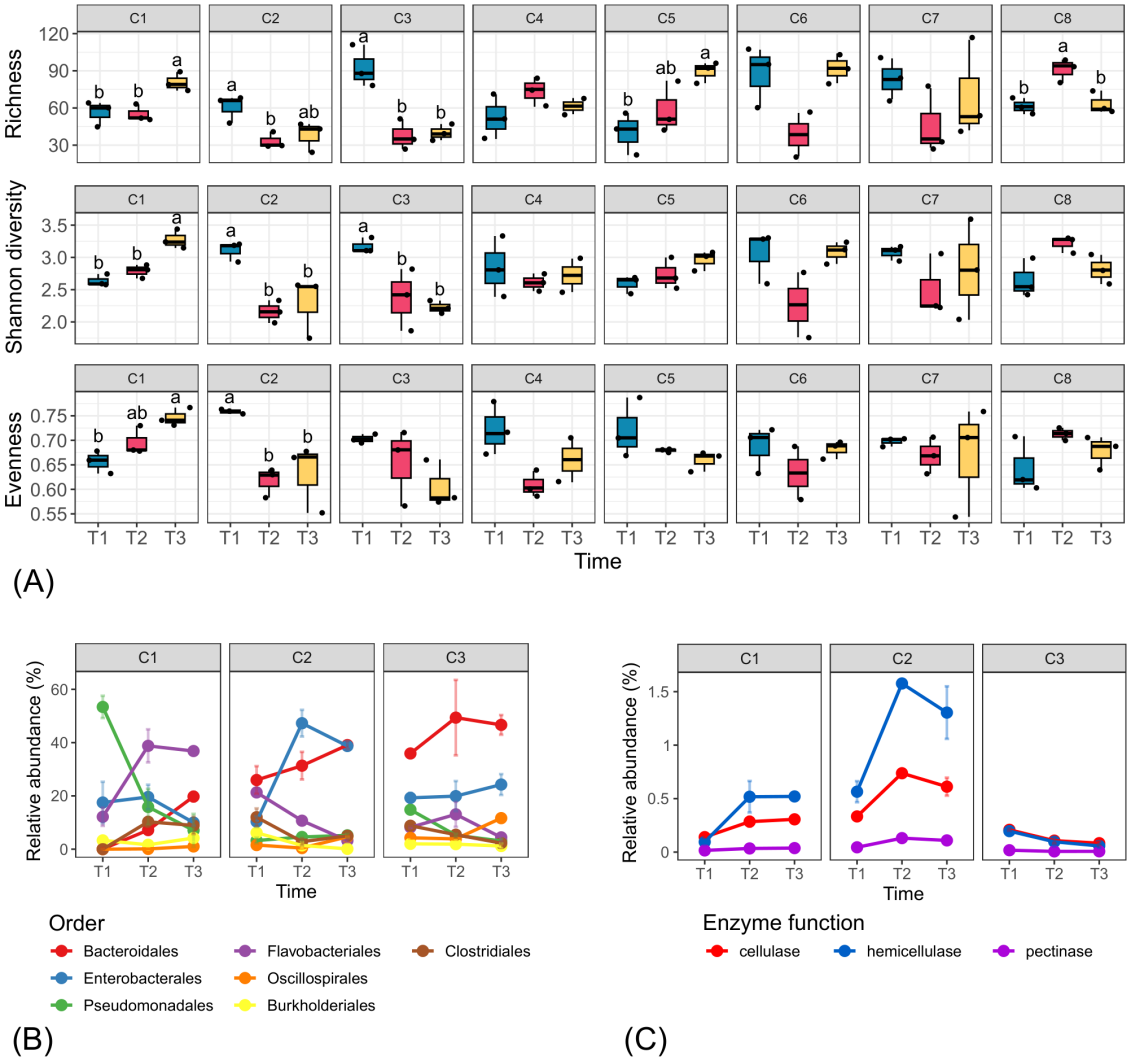
**(A)** Richness, Shannon diversity and evenness of the samples collected at different times in subsequent cycles. Within each cycle, significant differences between sampling times have been tested using Tukey HSD *post-hoc* test following ANOVA and are reported with letters. **(B)** Average relative abundances of the most abundant bacterial orders across the different sampling times in the first three retting cycles. Error bars represent the standard error. Different orders are shown in distinct colors. **(C)** Average relative abundances of the retting enzyme function guilds calculated over the total predicted enzyme function pool across the different sampling times in the first three retting cycles. Error bars represent the standard error. Different enzyme guilds are shown in distinct colors.

When examining community alpha diversity, fluctuations across the different sampling times were observed (**Figure 6A**), and these patterns were consistent among the investigated indices (richness, Shannon diversity and evenness). Overall, samples from T2 were characterized by lower values compared with T1 and T3. However, this difference was statistically significant only during the first two to three retting cycles for all the diversity indices.

As already reported in **Figures 3A**, **5A**, when considering the full dataset the different sampling times had only a limited effect on the bacterial community composition (PERMANOVA R^2^ = 0.07, p-value=0.002) or the predicted enzyme functions (PERMANOVA R^2^ = 0.03, p-value=non-significant); however, within-cycle analyses revealed a much stronger influence of the “sampling time” variable, especially in the earlier cycles (**Table 5**). This aspect was particularly evident on the predicted enzyme functions, where the sampling time had a strong and significant effect only in the first two cycles (C1–C2; R^2^ = 0.76–0.79, p-value<0.05).

**Table 5:** Effect of the “sampling time” variable (T1, T2, T3) on the bacterial community and the predicted retting-related enzyme functions within each cycle, tested using PERMANOVA.

Cycle	Bacteria		Enzymes	
	R^2^	p-value	R^2^	p-value
C1	0.74	0.008	0.76	0.04
C2	0.64	0.008	0.79	0.01
C3	0.59	0.01	0.57	n.s.
C4	0.58	0.006	0.50	n.s.
C5	0.56	0.008	0.14	n.s.
C6	0.56	0.006	0.30	n.s.
C7	0.49	0.02	0.26	n.s.
C8	0.47	0.01	0.27	n.s.

The magnitude of the effect (R^2^) and the significance (p-value) of the test are reported (n.s. = non-significant).

Focusing on the first three cycles, the inspection of the bacterial consortium composition at the order level across the different sampling times (T1, T2 and T3) revealed the three starting consortia to be quite different, and only few genera to be significantly modulated along the three sampling times (**Figure 6B**; **Supplementary Table 3**). The C1-T1 consortium was dominated by Pseudomonadales (53.4%) and Oscillospirales were undetectable; in C2-T1 the most abundant orders were Bacteroidales (26.3%) and Flavobacteriales (21.6%); in C3-T1 the most represented order was Bacteroidales (36.0%). Along the three sampling times of cycle C1, Pseudomonadales were significantly negatively selected, whereas the relative abundances of Flavobacteriales, Bacteroidales, Oscillospirales and Clostridiales increased significantly. Along the three sampling times of cycle C2, Enterobacterales and Oscillospirales were significantly positively selected, while Flavobacteriales and Burkholderiales significantly decreased. Along the three sampling times of C3, only Oscillospirales significantly increased, while Pseudomonadales and Clostridiales were significantly negatively selected. The other bacterial orders, although participating in the consortia, were not significantly selected along the three sampling times, based on the average of their relative abundances in the three sample replicates analyzed. Therefore, only Oscillospirales showed a consistent trend along the three sampling times of the analyzed retting cycles, being significantly positively selected during each cycle and thereof increasing their overall relative abundance in the retting consortium from undetectable to 11.7%. Furthermore, the relative abundances of Oscillospirales at T3 were particularly consistent with T1 of the subsequent cycle, indicating a continuum across the first three cycles (**Supplementary Table 3**).

Focusing on the relative abundances of the predicted enzyme functions relevant to the retting process (**Figure 6C**; **Supplementary Table 4**), a similar trend was observed for cellulases, hemicellulases, and pectinases in cycles C1 and C2, whereas cycle C3 displayed a distinct enzymatic pattern. More specifically, in the first two retting cycles most of the investigated enzyme functions showed a significant increase from sampling time T1 to T2–T3. In contrast, cycle C3 exhibited a general slight decrease in enzyme function abundance across the sampling times. However, as reported in **Table 5**, the effect of the “sampling time” variable on the retting-related enzymatic pool in cycle C3 was not significant (PERMANOVA R^2^ = 0.57, p-value=non-significant).

## 4 Discussion

Retting objectively represents a bottleneck along the straws processing line, substantially limiting the industrial production of quality hemp textile and technical fibers. The patented process developed at pilot scale into the prototype bioreactor and analyzed for microbial succession in the present study, could be an innovative alternative to conventional microbial retting techniques. Among the major outcomes of the developed technology are the drastic reduction of retting times (3 days) and water needs, and the less labor-intensive procedures compared to the traditional water retting method (Zimniewska, 2022; Bou Orm et al, 2024a). As traditional water retting, the total absence of additives characterizes the developed process. Furthermore, the containment within a bioreactor allows for greater control of the process, protecting the natural environmental microbiota from potential alterations. The experiment described in this paper was aimed at the study of the microbial community and its succession within the bioreactor for better control of the process and waste produced.

Retting liquor waste analysis confirmed to be quite similar to effluents of olive oil production, which can be re-used in agriculture as soil fertilizer, after careful analysis of its environmental impact (Fleyfel et al., 2022). The relatively low pH of retting waste would render it more suitable for calcareous soils, and the higher content of organic matter together with soil nutrients supports its possible reuse in fertilization programs, depending on soil structure, soil nutrition and crops. Nonetheless, further study is necessary to assess the absence of any phytotoxic activity and/or plant pathogenic bacteria which could prevent its use in agriculture.

### 4.1 Overall bacterial community composition and dynamics

Statistical analysis of the overall bacterial community composition revealed greater variability in the first three cycles, compared to subsequent ones. Furthermore, community composition appears to stabilize from cycle C4 onwards, becoming very similar in cycles C6-C8. These results are consistent with liquor recycling (5% of the retting liquor) from the second cycle onwards, and probably with the ability of process conditions to force the bacterial succession, reducing variability. Inspecting the co-occurrence networks, the number of edges, the networks diameters and the degree distribution suggested simplified and sparse microbial communities with limited competitive interactions in C1 and C2. Conversely, the denser connectivity, higher proportion of negative associations, and more heterogeneous degree distributions in later cycles could indicate increasingly complex and ecologically structured communities, that could be consistent with a transition toward more competitive and functionally differentiated assemblages. In terms of consortium efficiency, these results suggest the enrichment of the consortium with selected active bacteria along subsequent retting cycles.

Taxonomic classification of the 885 OTUs at the phylum level revealed the overall consortium to be mainly composed of Bacteroidota, Proteobacteria, and Firmicutes, as already found in different water retting systems of hemp straws by Fu et al. (2024) or flax, by Zhao et al. (2016), or kenaf, by Xu et al. (2022), and in dew retting of hemp straws by Ribeiro et al. (2015), Liu et al. (2017a), Law et al. (2020) and Bou Orm et al. (2023). A decrease in Proteobacteria relative abundance and an increase in Bacteroidota have been also observed along the eight subsequent cycles, according to Liu et al. (2017a), Law et al. (2020) and Bou Orm et al. (2023). These results support, at the phylum level, the existence of a core retting consortium (Bou Orm et al., 2024b) operating on different bast fiber species and retting systems, and a common trend of the bacterial succession in water retting and dew retting of hemp bast fiber. At the order level, nine taxa characterized the retting consortium developed in the bioreactor along subsequent cycles: Bacteroidales, Enterobacterales, Pseudomonadales, Flavobacteriales, Oscillospirales, Burkholderiales, Clostridiales, Sphingomonadales, and Lachnospirales with relative abundances significantly changing between subsequent cycles. All these taxa have been already found in the retting consortia operating on many bast fiber crops as hemp, flax, kenaf and jute (Djemiel et al., 2020; Bou Orm et al, 2024b).

Consortium temporal dynamics at the order level revealed the rapid succession within the first four cycles of the bacterial community and its relative stabilization around C5-C6. Based on their relative abundances, the retting consortium established during C1 was mainly composed by Flavobacteriales, Pseudomonadales and Enterobacterales. During C2, Bacteroidales and Enterobacterales equally dominated the retting consortium. Then, Bacteroidales gradually substituted Enterobacterales during C3, and from C4 onwards, Bacteroidales dominated the retting consortium. Along the eight retting cycles also a significant increase of Oscillospirales occurred, together with a significant decrease of Flavobacteriales, Pseudomonadales and Clostridiales. Enterobacterales and Burkholderiales relative abundances showed a fluctuating trend within the retting consortium, although their substantial different contribution to the consortium composition.

Bacteroidales are known to be widely involved in the retting process of different bast fiber crops, as they are able to grow and develop either on the surface of the bast fiber (Ribeiro et al., 2015) or in the water (Duan et al., 2020). Their increase in the retting consortium has been associated with the decrease in oxygen availability as they are known to be involved in the late stage of retting (Bou Orm et al., 2023; Aktar et al., 2024) due to their broader degrading activity especially of high molecular weight carbohydrates and proteins (Thomas et al., 2011). Among Bacteroidales, the most represented genus was *Bacteroides*, which drastically increased from C1 to C3 and dominated the consortium within C4-C8. Genomic analysis along the *Bacteroides* genus revealed some strains to carry specific Polysaccharide Utilization Loci (PULs) directly targeted to cellulose degradation, supporting their abundance in lignocellulose-degrading ecosystems (Naas et al., 2014).

Enterobacterales showed a fluctuating relative abundance within the retting consortium, often with an opposite trend with Pseudomonadales, which was more pronounced in the first three cycles. *Enterobacter* and *Escherichia-Shigella* were the most abundant genera within the Enterobacterales order*. Enterobacter* species play a significant role in the retting process, as part of the pectinolytic microbial community (Ray et al., 2022); accordingly, its relative abundance increased in the first four cycles and decreased from C5 to C8. From the perspective of liquor waste valorization as organic fertilizer, it is interesting to note that the *Enterobacter* genus has been known for its beneficial role on plant growth and productivity, therefore listed among the Plant Growth Promoting Rhizobacteria (PGPR) (Khalifa A., 2020). *Escherichia-Shigella* relative abundance significantly increased in the retting consortium from C6 to C8, according to Liu et al. (2017). Their cellulolytic activity under anaerobic conditions has been demonstrated by Wang et al. (2011), suggesting their detrimental impact on retted fiber quality within the retting consortium developing in the latest cycles.

Pseudomonadales are well known for their role in early phases of hemp, flax and jute microbial retting, due to their pectinolytic activity in aerobic and anaerobic conditions (Ribeiro et al., 2015; Zimniewska, 2022). However, they have been also observed to accumulate during the medium-late phase of jute water retting (Aktar et al., 2024). In addition, they can act on the degradation of lignin and hemicelluloses in plant fibers (Bou Orm et al., 2024a). The genera *Pseudomonas*, particularly known for its polygalacturonase and pectin lyase activity, and *Acinetobacter*, known for its pectinase activity (Zheng et al., 2011) were the most represented in the retting consortium developing within the pilot bioreactor, showing a coherent decreasing trend within C1-C3, and opposite within C4-C8. Both genera are aerobics, so their relative abundances fluctuations within the retting consortium could be related to oxygen availability and competition for the same substrates. The opposite trends of Enterobacterales and Pseudomonadales relative abundances within C1-C3 could be coherent with the prevalence of hemicellulase activity during the first retting cycles, which tends to equalize cellulase activity within C5, leading to a sort of coexistence of both activities from C6 onwards. Microbial species belonging to the genus *Pseudomonas* are known to play a significant role among PGPR due to their ability to promote plant growth, facilitating nutrient solubilization, biocontrol of some plant pathogens and bioremediation of organic and inorganic pollutants, including heavy metals (Das et al., 2020). The enrichment of *Pseudomonas* makes retting liquor waste particularly useful as organic fertilizer to support sustainable cropping systems.

Flavobacteriales order was mainly represented in the retting consortium by *Cloacibacterium*, *Flavobacterium* and *Chryseobacterium*, showing a reduction in their relative abundances across C1 to C8. Flavobacteriales belong to the CFB (Cytophaga-Flavobacterium-Bacteroides) group (classified under the Bacteroidota phylum) and comprise aerobic bacteria widely distributed in soil and freshwater, already known to have a significant role in retting communities (Munshi and Chattoo, 2008). As well as in jute water retting, they may act mainly when conditions are still partially aerobic or in transition towards anaerobic, according to their ability to metabolize pectins, xylose and arabinose (Aktar et al, 2024).

Oscillospirales, together with Clostridiales belong to the taxonomic class of Clostridia which are known to be obligate anaerobic bacteria, or micro-aerophiles. Along the eight subsequent retting cycles, their relative abundance significantly increased, and in particular that of *Caproiciproducens* and *Anaerophylum* genera. *Caproiciproducens* are non-spore-forming and strictly anaerobic bacteria producing acetic acid, butyric acid and caproic acid through the fermentation of diverse carbohydrates such as glycerol, l-arabinose, l-ribose, d-xylose, l-galactose, d-glucose, d-fructose, d-mannose, and also d-cellobiose (Jeon et al., 2023). It has been demonstrated that in systems optimizing cellulose-to-caproate production*, Caproiciproducens* often works alongside *Clostridium* species (Liu et al., 2026), so their increase in the retting consortium could be related to the increase of substrate availability produced along fiber retting. *Anaerofilum* species are obligate anaerobic bacteria, utilizing mono- and disaccharides such as glucose and xylose for their growth (Rainey, 2015) and their slight increase could be related to the gradual increase of substrates, and reduction of oxygen availability. An increase in some Clostridiales strains during bast fiber retting has been widely observed, as they are known to have pectinolytic activity (Tamburini et al., 2004; Ribeiro et al., 2015; Djemiel et al., 2020; Bou Orm et al., 2023, Aktar et al., 2024). However, in the pilot bioreactor the relative abundance of all the identified Clostridiales slightly decreases along C1 to C8, except for *Fonticella* members, which increased (**Supplementary Figure 1**). *Fonticella* is a newly identified genus within the Clostridiaceae family, and is strictly anaerobic, moderately thermophilic and halotolerant, living in natural hot springs and able to ferment cellobiose and other sugars, producing formate, acetate and ethanol (Fraj et al., 2013). The increasing trend along the subsequent cycles and the aquatic origin of this genus suggest that it enters the bioreactor *via* the spring water and is gradually enriched as fermentable substrates accumulate in the liquor.

Burkholderiales have been particularly described in the water retting consortia of kenaf bast fibers (Visi et al., 2013; Duan et al., 2020), or jute bast fibers (Munshi and Chattoo, 2008; Aktar et al., 2024), but also in dew retting of flax bast fibers (Djemiel et al., 2020). They showed a fluctuating trend within the retting consortium along C1 to C8. Among Burkholderiales, the genus *Acidovorax* dominated until C4, being substituted by *Aquitalea* and *Zoogloea* from C5-C8. *Alicycliphilus* and *Duganella* relative abundances remained relatively stationary during the eight retting cycles. *Acidovorax* and *Zoogloea* have been previously described as components of the anaerobic pectinolytic consortia developing in jute retting ponds (Munshi and Chattoo, 2008; Aktar et al., 2024). Among *Aquitalea*, high cellulolytic and ligninolytic activity have been reported within cellulose fermenting consortia (Woo et al., 2014). *Zoogloea* members are well known aerobic heterotrophic bacteria able to produce gelatinous colonies fluctuating within aqueous environment and implicated in floc formation with production of extracellular polymeric substances (Gao et al., 2018; Dahal et al., 2020). The increase in *Zoogloea* abundance in the retting community may be related to the progressive foam accumulation on the surface of the retting liquor, observed during the latest cycles. *Alicycliphilus* have been described in bacterial communities involved in wastewater treatment plants and exhibit a versatile metabolism under aerobic or anaerobic conditions (Solís-González and Loza-Tavera, 2019). *Duganella* are motile and aerobic bacteria, which are commonly isolated from soil and aquatic samples and are well-known for their antifungal effects (Haack et al., 2016). Their lignocellulolytic activities, including degradation of crystalline cellulose, xylanase activities, and lignin degrading activity, have been demonstrated (Maki et al., 2012).

### 4.2 Trends of predicted retting enzyme functions across retting cycles

Overall, predicted retting-related enzyme function abundances within the bacterial consortium developed across C1-C8 cycles highlight the dominance of cellulase over hemicellulase and the relative low representation of pectinase functions. These results could correlate with the observation that pectinolytic strains could be anaerobics (like *Clostridium* spp) and aerobics (like *Bacillus* spp) (Tamburini et al., 2004) but in the retting consortium resulting from metabarcoding data, aerobic pectinolytic bacteria like *Bacillus* strains were not found to be enriched during C1-C8. Therefore, pectinolytic activity could be more represented within bast fibers than in the retting liquor, where oxygenation is guaranteed by air insufflation. The PCoA of predicted enzyme functions composition (**Figure 5A**) highlighted a temporal shift of the selected functions within the retting consortium, as already shown for taxa composition, confirming retting functional consortium to follow the same temporal dynamics of the bacterial consortium. In terms of predicted consortium efficiency, the summarized enzymatic guilds for cellulase, hemicellulase and pectinase functions across the eight retting cycles highlighted the synergic relationship existing between these functions during retting and confirmed what postulated, based on bacterial succession. It is well known that hemicellulases have a pivotal role in degrading the hemicellulose matrix surrounding the cellulose fibrils, making the latter accessible to the action of cellulases. On the other hand, an excess of cellulase activity during retting is considered detrimental for the technical properties of the retted fibers (Liu et al., 2017a). Based on functional predictions, the retting consortium appeared to increase its efficiency during the first two cycles, reaching the highest relative abundance of retting-related functions, as well as the best hemicellulase-to-cellulase function ratio, during C2. Then, from C3 onward, consortium activity turned, due to an increase in cellulase functions, remaining nearly stable until C6. Starting from C6, the excess of cellulase over hemicellulase functions and the slight reduction of pectinase functions, could indicate the establishment of an over-retting condition (Paridah et al., 2011). So, liquor bacterial consortium seemed to become more complex in later cycles, suggesting the gradual establishment of a stable and active retting community. On the other hand, the abundances of predicted enzyme functions point to an increased efficiency in cellulose degradation during the late cycles, characteristic of over-retting conditions.

### 4.3 Temporal dynamics of the retting consortium within retting cycles

When analyzing the effect of sampling time (T1, T2, T3) on the bacterial diversity along C1-C8 cycles, significant differences in diversity indices between different sampling times have been generally observed only for the first three cycles. A comparative analysis of the bacterial taxa and selected predicted functions, significantly changing within the first three cycles, highlighted the functional dynamics occurring at the same time within the retting liquor. Based on taxa relative abundances, a substantial difference has been observed between the composition of the three consortia at T1 of the first three cycles. This data is consistent with the different contributions to the retting consortium of the bast fibers (belonging to different hemp varieties from different cultivation areas), spring water, and inoculum (5%) in C2 and C3. However, starting from different consortia, only Oscillospirales followed a significant and consistent trend along the three sampling times of the three subsequent cycles, showing a constant positive selection across the three cycles. This selection resulted in the overall increase of Oscillospirales relative abundance along a continuum, from 0% (undetectable) to 11.7% across C1-C3.

Enzyme functions predictions at the same sampling time across C1-C3 revealed a common pattern between C1 and C2 represented by the significant increase at T2 of all the enzyme functions investigated. This pattern was not confirmed in C3 where a slight decrease in all enzymatic functions related to retting has been found. These observations are consistent with the rapid establishment of an efficient retting community within the first two cycles, probably due to the recycling of 5% of the retting liquor, but also indicates as rapidly its efficiency is reduced during the subsequent cycles, maybe due to the reduction of available substrates.

## 5 Conclusions

Innovation of fiber retting is considered one of the major challenges for the full exploitation of natural bast fibers into sustainable textiles or constructions. The process developed at pilot scale could be a valuable alternative to traditional water retting, due to the drastic reduction of retting times and water needs, without using additives. DNA metabarcoding of the retting liquor, together with enzyme functions predictions across eight consecutive retting cycles, revealed a successional pattern resembling that generally observed in natural retting ponds during long-term fiber incubation. It also highlighted how rapidly this community structure becomes established in the earliest cycles. However, the processes occurring in the retting liquor may not fully mirror those taking place on the fiber itself, due to the distinct nature of the two substrates (liquid versus solid) and their different contribution to the retting consortium. The negative impact of liquor recycling on retting-related enzyme function abundances over the third retting cycle has been measured, as well as the possible magnification potential of the bioreactor of bacterial genera introduced with spring water (e.g., *Fonticella*). Useful information has been obtained on possible further valorization of liquor wastes as organic fertilizer, due to the enrichment across subsequent retting cycles of organic matter and nutrients, but also of bacterial taxa, such as *Enterobacter* and *Pseudomonas*, listed among Plant Growth Promoting Rhizobacteria (PGPR). Anyway, a more accurate study of the impact on crop and soil health will be necessary to assess if retting liquor waste could be actually used in sustainable agricultural practices. Finally, further insights into the developed technology will derive from the investigation of microbial succession (bacteria and fungi) occurring on bast fibers throughout the retting cycles.

## Data Availability

The datasets presented in this study can be found in online repositories. The names of the repository/repositories and accession number(s) can be found below: https://www.ncbi.nlm.nih.gov/, PRJNA1453432.
