# Supplementary material for: A prototype bioreactor for the water retting of hemp bast fiber: dynamics of the liquor bacterial consortium across sequential retting cycles
Source: Front Plant Sci. 2026 Aug 5;17:1856675. doi: 10.3389/fpls.2026.1856675 (PMC13486293; doi:10.3389/fpls.2026.1856675)
Supplement: Supplementary file 1 [file DataSheet1.docx]

Supplementary Material

**Supplementary Figure 1.** Scheme of the hemp fiber retted within the prototype bioreactor further processed into 100% hemp yarns (courtesy of Prototipo Studio – Lampoon Magazine).

**Supplementary Figure 2.** Rarefaction curves of all samples, generated after rarefaction to 5,443 reads per sample.

**Supplementary Table 1.** Average percentages (%) of the most abundant bacterial phyla and orders within each retting cycle. Differences in the phyla/orders absolute abundance among the different cycles were tested using ANOVA, and significant differences (p-value < 0.05) are evidenced in bold and reported with different letters.

| **Cycle** | **Bacteroidota** | **Proteobacteria** | **Firmicutes** | **Bacteroidales** | **Enterobacterales** | **Pseudomonadales** | **Flavobacteriales** | **Oscillospirales** | **Burkholderiales** | **Clostridiales** |
| --- | --- | --- | --- | --- | --- | --- | --- | --- | --- | --- |
| C1 | 39.3 | 49.6 | **11.0 (bc)** | **9.06 (b)** | **15.7 (b)** | **25.5 (a)** | **29.4 (a)** | **0.39 (c)** | **3.06 (ab)** | **6.43 (a)** |
| C2 | 45.8 | 42.1 | **12.1 (bc)** | **32.4 (a)** | **32.4 (a)** | **4.38 (b)** | **11.8 (b)** | **2.24 (c)** | **2.60 (ab)** | **6.68 (a)** |
| C3 | 52.7 | 33.1 | **14.1 (abc)** | **44.0 (a)** | **21.2 (ab)** | **7.72 (b)** | **8.58 (bc)** | **6.60 (bc)** | **1.70 (b)** | **5.51 (ab)** |
| C4 | 50.3 | 38.0 | **11.6 (bc)** | **45.8 (a)** | **10.9 (b)** | **14.9 (ab)** | **3.38 (bc)** | **6.01 (bc)** | **6.59 (ab)** | **3.16 (ab)** |
| C5 | 49.9 | 41.7 | **8.41 (c)** | **41.8 (a)** | **14.2 (b)** | **14.1 (ab)** | **7.20 (bc)** | **5.50 (bc)** | **4.75 (ab)** | **1.10 (b)** |
| C6 | 45.4 | 38.2 | **16.2 (abc)** | **42.5 (a)** | **9.78 (b)** | **14.0 (ab)** | **2.70 (bc)** | **9.71 (ab)** | **7.25 (a)** | **2.17 (ab)** |
| C7 | 47.6 | 32.7 | **19.5 (ab)** | **45.9 (a)** | **11.6 (b)** | **12.5 (ab)** | **1.41 (c)** | **9.45 (ab)** | **4.74 (ab)** | **2.68 (ab)** |
| C8 | 43.2 | 32.2 | **24.3 (a)** | **43.2 (a)** | **17.8 (b)** | **8.78 (ab)** | **0.38 (c)** | **14.7 (a)** | **3.54 (ab)** | **4.95 (ab)** |

**Supplementary Figure 3.** Average relative abundances of the bacterial genera belonging to the most abundant orders across cycles. Error bars represent the standard error. Only genera showing a clear trend across successive cycles are displayed. Different genera are shown in distinct colors.

**Supplementary Table 2.** Average percentages (%) of the retting enzyme functions and enzymatic guilds, calculated over the total predicted enzyme function pool, within each retting cycle. Differences in the enzyme functions absolute abundance among the different cycles were tested using ANOVA, and significant differences (p-value < 0.05) are evidenced in bold and reported with different letters.

| **Cycle** | **Cellulases** | **EC:3.2.1.21** | **EC:3.2.1.4** | **EC:3.2.1.91** | **Hemicellulases** | **EC:3.2.1.23** | **EC:3.2.1.51** | **EC:3.2.1.55** | **EC:3.2.1.177** | **EC:3.2.1.78** | **EC:3.2.1.25** | **EC:3.2.1.8** | **EC:3.2.1.37** | **Pectinases** | **EC:3.1.1.11** | **EC:3.1.1.73** | **EC:4.2.2.23** | **EC:4.2.2.10** | **EC:4.2.2.2** |
| --- | --- | --- | --- | --- | --- | --- | --- | --- | --- | --- | --- | --- | --- | --- | --- | --- | --- | --- | --- |
| C1 | **0.244 (b)** | **0.197 (b)** | **0.046 (b)** | <0.001 | **0.378 (b)** | **0.166 (b)** | **0.111 (b)** | **0.038 (b)** | **0.017 (b)** | **0.022 (b)** | **0.016 (b)** | **0.007 (a)** | 0.001 | **0.029 (b)** | **0.026 (b)** | 0.002 | <0.001 | **<0.001 (b)** | <0.001 |
| C2 | **0.561 (a)** | **0.523 (a)** | **0.038 (b)** | 0 | **1.149 (a)** | **0.479 (a)** | **0.335 (a)** | **0.142 (a)** | **0.049 (a)** | **0.092 (a)** | **0.048 (a)** | **0.001 (b)** | 0.002 | **0.095 (a)** | **0.093 (a)** | 0.002 | 0 | **0 (b)** | 0 |
| C3 | **0.133 (b)** | **0.100 (b)** | **0.034 (b)** | 0 | **0.117 (b)** | **0.062 (b)** | **0.026 (b)** | **0.010 (b)** | **0.008 (b)** | **0.005 (b)** | **0.004 (b)** | **0.002 (b)** | <0.001 | **0.010 (b)** | **0.010 (b)** | <0.001 | 0 | **<0.001 (b)** | <0.001 |
| C4 | **0.159 (b)** | **0.095 (b)** | **0.064 (ab)** | 0 | **0.127 (b)** | **0.058 (b)** | **0.028 (b)** | **0.013 (b)** | **0.007 (b)** | **0.006 (b)** | **0.008 (b)** | **0.005 (ab)** | 0.001 | **0.011 (b)** | **0.010 (b)** | 0.001 | 0 | **<0.001 (b)** | <0.001 |
| C5 | **0.218 (b)** | **0.169 (b)** | **0.049 (b)** | 0 | **0.253 (b)** | **0.107 (b)** | **0.070 (b)** | **0.027 (b)** | **0.015 (b)** | **0.017 (b)** | **0.014 (b)** | **0.004 (ab)** | 0.001 | **0.021 (b)** | **0.020 (b)** | 0.001 | <0.001 | **<0.001 (b)** | <0.001 |
| C6 | **0.134 (b)** | **0.093 (b)** | **0.041 (b)** | 0 | **0.082 (b)** | **0.036 (b)** | **0.025 (b)** | **0.005 (b)** | **0.006 (b)** | **0.001 (b)** | **0.004 (b)** | **0.002 (b)** | 0.002 | **0.008 (b)** | **0.006 (b)** | 0.002 | <0.001 | **<0.001 (b)** | <0.001 |
| C7 | **0.196 (b)** | **0.131 (b)** | **0.064 (ab)** | <0.001 | **0.115 (b)** | **0.055 (b)** | **0.028 (b)** | **0.011 (b)** | **0.006 (b)** | **0.005 (b)** | **0.004 (b)** | **0.005 (ab)** | 0.001 | **0.008 (b)** | **0.006 (b)** | 0.001 | 0.001 | **0.001 (a)** | <0.001 |
| C8 | **0.208 (b)** | **0.123 (b)** | **0.086 (a)** | <0.001 | **0.124 (b)** | **0.057 (b)** | **0.033 (b)** | **0.011 (b)** | **0.008 (b)** | **0.004 (b)** | **0.003 (b)** | **0.008 (a)** | 0.001 | **0.008 (b)** | **0.007 (b)** | 0.001 | <0.001 | **<0.001 (ab)** | <0.001 |

**Supplementary Table 3.** Average percentages (%) of the most abundant bacterial orders within each sampling time of the first three retting cycles. Differences in the orders absolute abundance among the different sampling times within each cycle were tested using ANOVA, and significant differences (p-value < 0.05) are evidenced in bold and reported with different letters.

| **Cycle** | **Time** | **Bacteroidales** | **Enterobacterales** | **Pseudomonadales** | **Flavobacteriales** | **Oscillospirales** | **Burkholderiales** | **Clostridiales** |
| --- | --- | --- | --- | --- | --- | --- | --- | --- |
| C1 | T1 | **0.09 (c)** | 17.5 | **53.4 (a)** | **12.2 (b)** | **0 (b)** | 3.30 | **0.01 (b)** |
|  | T2 | **7.19 (b)** | 19.6 | **15.9 (b)** | **38.9 (a)** | **0.11 (b)** | 1.66 | **10.3 (a)** |
|  | T3 | **19.9 (a)** | 10.1 | **7.12 (b)** | **37.1 (a)** | **1.03 (a)** | 4.23 | **8.99 (ab)** |
| C2 | T1 | 26.3 | **10.5 (b)** | 3.29 | **21.6 (a)** | **1.57 (b)** | **6.25 (a)** | 12.2 |
|  | T2 | 31.3 | **47.3 (a)** | 4.57 | **10.7 (b)** | **0.42 (c)** | **1.41 (b)** | 2.82 |
|  | T3 | 39.6 | **39.3 (a)** | 5.27 | **3.25 (c)** | **4.27 (a)** | **0.13 (b)** | 5.05 |
| C3 | T1 | 36.0 | 19.3 | **14.8 (a)** | 8.22 | **4.27 (b)** | 2.01 | **8.81 (a)** |
|  | T2 | 49.4 | 19.9 | **5.23 (b)** | 13.1 | **3.86 (b)** | 1.89 | **5.43 (ab)** |
|  | T3 | 46.7 | 24.3 | **3.11 (b)** | 4.44 | **11.7 (a)** | 1.19 | **2.29 (b)** |

**Supplementary Table 4.** Average percentages (%) of the retting enzyme functions and enzymatic guilds, calculated over the total predicted enzyme function pool, within each sampling time of the first three retting cycles. Differences in the enzyme functions absolute abundance among the different sampling times within each cycle were tested using ANOVA, and significant differences (p-value < 0.05) are evidenced in bold and reported with different letters.

| **Cycle** | **Time** | **Cellulases** | **EC:3.2.1.21** | **EC:3.2.1.4** | **EC:3.2.1.91** | **Hemicellulases** | **EC:3.2.1.23** | **EC:3.2.1.51** | **EC:3.2.1.55** | **EC:3.2.1.177** | **EC:3.2.1.78** | **EC:3.2.1.25** | **EC:3.2.1.8** | **EC:3.2.1.37** | **Pectinases** | **EC:3.1.1.11** | **EC:3.1.1.73** | **EC:4.2.2.23** | **EC:4.2.2.10** | **EC:4.2.2.2** |
| --- | --- | --- | --- | --- | --- | --- | --- | --- | --- | --- | --- | --- | --- | --- | --- | --- | --- | --- | --- | --- |
| C1 | T1 | **0.140 (b)** | **0.098 (b)** | 0.042 | 0 | **0.095 (b)** | **0.042 (b)** | **0.020 (b)** | **0.007 (b)** | 0.010 | **0.003 (b)** | **0.004 (b)** | 0.009 | 0.002 | 0.016 | 0.009 | 0.004 | 0.002 | 0 | 0.001 |
|  | T2 | **0.285 (a)** | **0.248 (a)** | 0.036 | <0.001 | **0.518 (a)** | **0.231 (a)** | **0.159 (a)** | **0.054 (a)** | 0.020 | **0.031 (ab)** | **0.019 (ab)** | 0.002 | 0.001 | 0.034 | 0.033 | 0.001 | 0 | <0.001 | <0.001 |
|  | T3 | **0.306 (a)** | **0.246 (a)** | 0.061 | 0 | **0.520 (a)** | **0.224 (a)** | **0.153 (a)** | **0.054 (a)** | 0.021 | **0.034 (a)** | **0.024 (a)** | 0.010 | 0.001 | 0.037 | 0.037 | 0.001 | <0.001 | <0.001 | 0 |
| C2 | T1 | **0.334 (b)** | **0.292 (b)** | 0.041 | 0 | **0.565 (b)** | **0.230 (b)** | **0.169 (b)** | **0.069 (b)** | **0.026 (b)** | **0.039 (b)** | **0.023 (b)** | **0.004 (a)** | 0.005 | **0.045 (b)** | **0.039 (b)** | 0.006 | 0 | 0 | 0 |
|  | T2 | **0.738 (a)** | **0.702 (a)** | 0.036 | 0 | **1.577 (a)** | **0.659 (a)** | **0.459 (a)** | **0.197 (a)** | **0.065 (a)** | **0.131 (a)** | **0.066 (a)** | **0 (b)** | 0 | **0.131 (a)** | **0.131 (a)** | 0 | 0 | 0 | 0 |
|  | T3 | **0.612 (a)** | **0.574 (a)** | 0.038 | 0 | **1.304 (a)** | **0.548 (a)** | **0.377 (a)** | **0.162 (a)** | **0.056 (a)** | **0.107 (a)** | **0.054 (a)** | **<0.001 (b)** | 0.001 | **0.109 (a)** | **0.109 (a)** | 0.001 | 0 | 0 | 0 |
| C3 | T1 | **0.209 (a)** | **0.156 (a)** | 0.053 | 0 | **0.196 (a)** | **0.105 (a)** | 0.044 | **0.017 (a)** | **0.013 (a)** | **0.010 (a)** | **0.006 (a)** | 0.002 | <0.001 | **0.018 (a)** | **0.017 (a)** | <0.001 | 0 | 0 | <0.001 |
|  | T2 | **0.097 (b)** | **0.081 (b)** | 0.026 | 0 | **0.097 (ab)** | **0.049 (b)** | 0.026 | **0.007 (b)** | **0.004 (b)** | **0.004 (ab)** | **0.003 (b)** | 0.004 | <0.001 | **0.007 (b)** | **0.006 (b)** | <0.001 | 0 | <0.001 | 0 |
|  | T3 | **0.058 (b)** | **0.062 (b)** | 0.022 | 0 | **0.058 (b)** | **0.032 (b)** | 0.009 | **0.005 (b)** | **0.007 (b)** | **0.002 (b)** | **0.001 (b)** | 0.001 | 0.001 | **0.007 (b)** | **0.007 (b)** | 0.001 | 0 | 0 | <0.001 |
